# Chemical and molecular bases of dome formation in human colorectal cancer cells mediated by sulphur compounds from *Cucumis melo* var. *conomon*

**DOI:** 10.1002/2211-5463.13001

**Published:** 2020-11-06

**Authors:** Miyu Kamimura, Azusa Sasaki, Shimpei Watanabe, Shiho Tanaka, Akiko Fukukawa, Kazuya Takeda, Yasushi Nakamura, Takako Nakamura, Kouji Kuramochi, Yui Otani, Fumio Hashimoto, Kanji Ishimaru, Tomoaki Matsuo, Shigehisa Okamoto

**Affiliations:** ^1^ Laboratory of Plant Life Science and Technology United Graduate School of Agricultural Sciences Kagoshima University Kagoshima Japan; ^2^ Laboratory of Food Science Graduate School of Life and Environmental Sciences Kyoto Prefectural University Kyoto Japan; ^3^ Department of Japanese Food Culture Faculty of Letters Kyoto Prefectural University Kyoto Japan; ^4^ Department of Applied Biological Science Tokyo University of Science Tokyo Japan; ^5^ Department of Applied Biological Sciences Faculty of Agriculture Saga University Saga Japan

**Keywords:** CCNE2, CDC25A, colorectal cancer, differentiation, dome, methylthioacetic acid

## Abstract

Colorectal cancer was the third most commonly diagnosed malignant tumor and the fourth leading cause of cancer deaths worldwide in 2012. A human colorectal cancer cell line, RCM‐1, was established from a colon cancer tissue diagnosed as a well‐differentiated rectum adenocarcinoma. RCM‐1 cells spontaneously form ‘domes’ (formerly designated ‘ducts’) resembling villiform structures. Two sulphur‐containing compounds from *Cucumis melo* var. *conomon* (Katsura‐uri, or Japanese pickling melon), referred to as 3‐methylthiopropionic acid ethyl ester (MTPE) and methylthioacetic acid ethyl ester (MTAE), can induce the differentiation of the unorganized cell mass of an RCM‐1 human colorectal cancer cell culture into a dome. However, the underlying molecular mechanisms of such dome formation have not been previously reported. Here, we performed a structure–activity relationship analysis, which indicated that methylthioacetic acid (MTA) was the lowest molecular weight compound with the most potent dome‐inducing activity among 37 MTPE and MTAE analogues, and the methylthio group was essential for this activity. According to our microarray analysis, MTA resulted in down‐regulation of 537 genes and up‐regulation of 117 genes. Furthermore, MTA caused down‐regulation of many genes involved in cell‐cycle control, with the *cyclin E2* (*CCNE2*) and *cell division cycle 25A* (*CDC25A*) genes being the most significantly reduced. Pharmacological analysis showed that the administration of two cell‐cycle inhibitors for inactivating CDC25A phosphatase (NSC95397) and the cyclin E2/cyclin‐dependent kinase 2 complex (purvalanol A) increased the dome number independently of MTA. Altogether, our results indicate that MTA is the minimum unit required to induce dome formation, with the down‐regulation of *CDC25A* and possibly *CCNE2* being important steps in this process.

## Graphical abstract

This study showed that methylthioacetic acid (MTA), an analogue of the sulphur‐containing phytochemicals isolated from *Cucumis melo* var. *conomon*, most potently differentiated RCM‐1 colon cancer cells into domes. MTA was shown to strongly down‐regulate *CDC25A* and *CCNE2*, and their inhibitors prompted dome formation independently of MTA, suggesting that MTA may induce dome formation through dysfunction of these G1 regulators.

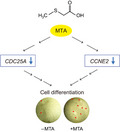

**Abbreviations**
ALP: alkaline phosphatase
CCNE2: cyclin E2
CDC25A: cell division cycle 25A
CDK: cyclin‐dependent kinase
GAPDH: glyceraldehyde 3‐phosphate dehydrogenase
GO: Gene Ontology
GSEA: gene set enrichment analysis
KEGG: Kyoto Encyclopedia of Genes and Genomes
MTA: methylthioacetic acid
MTAE: methylthioacetic acid ethyl ester
MTPE: 3‐methylthiopropionic acid ethyl ester

Colorectal cancer was the third most commonly diagnosed malignant tumor and the fourth leading cause of cancer deaths worldwide in 2012 [1]. Furthermore, because colorectal cancer is known as a ‘silent’ disease that may show minimal or even no symptoms until the later stages (i.e. stage II and beyond), its diagnosis at the early stage is difficult [2, 3]. Surgical excision combined with follow‐up chemotherapy to kill the remaining tumor cells in the body has undoubtedly been significant for curing colorectal cancers and preventing their recurrence [4]; however, the toxicity of the chemotherapeutic agents toward the vigorously dividing normal cells has led to many patients suffering detrimental side effects [5, 6]. Therefore, apart from the classical cancer therapies, other chemotherapies without the agents for massacring cells are urgently needed. One promising alternative is differentiation therapy, which is defined as a pharmacological therapeutic that directs tumor cells toward their differentiation, maturation or senescence, thereby alleviating the cancer symptoms [7]. Because this therapy can convert tumor tissue into morphologically differentiated tissue without killing the normal noncancerous cells, the adverse side effects may be reduced. In addition, the treatment is theoretically applicable to patients with the later stages of cancer and may thus help them to maintain a relatively high quality of life. Accordingly, differentiation therapy in combination with the already established methods would have great potential in cancer treatment.

A human colorectal cancer cell line, RCM‐1, has been established from a colon cancer tissue diagnosed as a well‐differentiated rectum adenocarcinoma [8]. The RCM‐1 cells spontaneously form ‘domes’ (formerly designated ‘ducts’) resembling villiform structures [9]. The domes are 3D multicellular structures caused by vectorial fluid transport from apical surface to basolateral surface, leading to fluid accumulation in a localized area between the monolayer and the surface of the culture flask [10, 11], and often observed on cell cultures derived from tumors (lung adenocarcinoma, mammary adenocarcinoma and colonic adenocarcinoma), as well as normal tissues (kidney epithelium) [10, 12, 13]. In addition, the domes are reportedly formed in response to known differentiation inducers [14, 15]. Thus, to date, dome formation has been used as a morphological marker in the cell cultures for the onset of cell differentiation for studying transepithelial transport and cell polarity, because that unidirectional fluid movement mimics the active transepithelial transport seen in differentiated epithelial tissues of colon and kidney [10, 11, 16, 17, 18].

Plants are a rich source of natural compounds with different biological activities because they produce a huge variety of phytochemicals (plant secondary metabolites) that have diverse and advantageous roles in the increased health and survival of plants, and that are also beneficial for disease control in humans and animals due to pharmacological activity [19]. Indeed, nearly 50% of the anticancer agents approved since the 1940s are natural compounds *per se*, being semisynthesized or completely synthesized analogues of natural biologicals, and they also include phytochemical‐derived compounds, such as vincristine and homoharringtonine [20, 21]. In line with this fact, we previously screened phytochemicals with biological abilities from fully ripened fruits of an heirloom vegetable, Katsura‐uri (*Cucumis melo* var. *conomon*), using dome formation as a guide of purification, and isolated 3‐methylthiopropionic acid ethyl ester (MTPE) and methylthioacetic acid ethyl ester (MTAE) [9, 22]. Furthermore, MTPE was shown to induce alkaline phosphatase (ALP) activity as a biochemical differentiation marker in RCM‐1 cells [9]. Because they can efficiently convert unorganized cell mass into the domes, MTPE and MTAE conceivably have an ability to induce the differentiation of the RCM‐1 human colorectal cancer cell line. However, the molecular mechanisms underlying the dome formation induced by MTPE and MTAE remain unknown.

In this study, we conducted a structure–activity relationship analysis to determine the minimum unit and indispensable moiety of MTPE‐ and MTAE‐related compounds required to form domes in RCM‐1 cell culture. Then we found that methylthioacetic acid (MTA) was the lowest molecular weight compound with the most potent activity in this regard, and that the methylthio group was essential for its activity. Furthermore, we carried out microarray and pharmacological analyses to elucidate the underlying mechanisms of the MTA‐induced dome formation and determined the involvement of the cell division cycle 25A (CDC25A) phosphatase and possibly cyclin E2 (CCNE2), both G1 regulators of cell‐cycle control, in the MTA pathway.

## Materials and methods

### Chemicals

MTA was obtained from Matrix Scientific (Elgin, SC, USA), whereas MTPE and MTAE were purchased from Avocado Research Chemicals, Ltd. (Heysham, Lancashire, UK). Methoxyacetic acid, 3‐methylthiopropionic acid and methylthioacetic acid methyl ester were obtained from Tokyo Chemical Industry Co., Ltd. (Tokyo, Japan). Unless otherwise noted, all other chemicals were purchased from Fujifilm Wako Pure Chemical Corporation (Osaka, Japan). The cell‐cycle inhibitors purvalanol A and NSC95397 were purchased from Cosmo Bio Co., Ltd. (Tokyo, Japan) and Merck KGaA (Darmstadt, Germany), respectively. The following compounds of >95% purity that were used in the structure–activity relationship analyses were a gift from the Laboratory of Synthetic Chemistry of Functional Molecules, Graduate School of Life and Environmental Sciences, Kyoto Prefectural University: mercaptoacetic acid ethyl ester, mercaptoacetic acid propyl ester, mercaptoacetic acid butyl ester, mercaptopropionic acid ethyl ester, mercaptopropionic acid methyl ester, mercaptopropionic acid propyl ester, methylthioacetic acid propyl ester, 3‐methylthiopropionic acid propyl ester, 4‐methylthiobutyric acid, 4‐methylthiobutyric acid methyl ester, 4‐methylthiobutyric acid ethyl ester, 5‐methylthiovaleric acid, 5‐methylthiovaleric acid methyl ester, 5‐methylthiovaleric acid ethyl ester, ethylthioacetic acid ethyl ester, ethylthioacetic acid methyl ester, ethylthioacetic acid propyl ester, 3‐ethylthiopropionic acid, 3‐ethylthiopropionic acid methyl ester, 3‐ethylthiopropionic acid ethyl ester, 4‐ethylthiobutyric acid, 4‐ethylthiobutyric acid methyl ester, 4‐ethylthiobutyric acid ethyl ester, propylthioacetic acid, propylthioacetic acid methyl ester, 3‐propylthiopropionic acid and 3‐propylthiopropionic acid ethyl ester.

### Cell culture

RCM‐1 cells were obtained from Japanese Collection of Research Bioresources Cell Bank (JCRB0256, RRID: CVCL_1648). Ehrlich cells were purchased from RIKEN Cell Bank (RCB0142, RRID: CVCL_3873, Tsukuba, Ibaraki, Japan). WB‐F344 and CoCM‐1 cells were kindly gifted by J.W. Grisham and M.S. Tsao of University of North Carolina (USA) and H. Kataoka of University of Miyazaki (Japan), respectively. HCT116 cells were a kind gift from M. Brattain (Baylor College of Medicine, USA). WB‐Ha‐ras, WB‐neu, WBsrc‐neo2, WB‐myc/ras, MSU‐2 and Ming cells were obtained from Michigan State University (USA). BxPC3, HaPT1 and HPD1NR cells were obtained from Nara Medical University (Japan). KNC cells were established by Y. Nakamura of Kyoto Prefectural University (Japan) [23]. RCM‐1, Ehrlich, CoCM‐1, BxPC3, HaPT1 and HPD1NR cells were cultured in a medium made up of equal volumes of RPMI 1640 and Ham’s F12 (Life Technologies, Carlsbad, CA, USA) and supplemented with 10% FBS (Biowest, Nuaillé, France) and 10 μg·mL^−1^ gentamicin (Thermo Fisher Scientific, Waltham, MA, USA). WB‐Ha‐ras cells were grown in modified Eagle’s medium (Gibco, Grand Island, NY, USA) supplemented with 5% FBS. WB‐neu and WB‐myc/ras cells were incubated in modified Eagle's medium supplemented with 7% FBS and 50 μg·mL^−1^ gentamicin. KNC cells were grown in defined keratinocyte serum free medium (Gibco) containing 10% FBS. The remaining cells were cultured in modified Eagle's medium supplemented with 10% FBS. All cells were cultured at 37 °C in 5% CO_2_ atmosphere.

### Dome formation assay

The dome formation assay for evaluating the ability of the different chemicals to induce domes in RCM‐1 cell culture was carried out according to a previously described method [22]. In brief, RCM‐1 cells (1.0 × 10^5^ or 1.5 × 10^5^) were seeded into 96‐well plates and cultured to near confluency, after which they were treated with different concentrations of the chemical compounds for 48 h. Thereafter, the number of domes formed was counted under a ZEISS Axiovert 25 microscope (Oberkochen, Germany). The ED_50_ value was used as an index to estimate the activity of the chemicals, where it was defined as the lowest dose (mm) at which the dome number was enhanced by more than 1.5‐fold compared with that of the mock control.

### ALP activity

ALP activity was measured by LabAssay™ ALP kit according to the supplier's instructions (Fujifilm Wako Pure Chemical Corporation). In brief, after two washes with PBS, cells were lysed with the help of sonication for 10 s in a buffer containing 0.1 m carbonate, 2 mm MgCl_2_ (pH 9.8) and 0.3% Nonidet P‐40. Cell lysates were applied to ALP reaction using p‐nitrophenylphosphate, an ALP substrate, for 15 min at 37 °C, followed by measuring the absorbance at 405 nm (*A*_405 nm_) with the microplate reader (Mithras LB 940; Berthold Technologies GmbH & Co.KG, Bad Wildbad, Germany). Protein concentration in the lysate was determined using Protein Assay Bradford Reagent (Fujifilm).

### MTA treatment

One millimolar of MTA was applied to nearly confluent RCM‐1 cells for different periods, which were determined dependent on each analysis. In the microarray, RT‐PCR and immunoblot analyses, following incubation with MTA, the MTA‐containing medium was replaced with the corresponding vehicle medium, and the cells were further cultured until cell harvesting. As a control, acetonitrile (the solvent used to dissolve MTA) was applied to the cells at the final concentration of 0.1%. The dome number was checked every time to evaluate the effectiveness of MTA in each experiment.

### Microarray analysis

The microarray analysis for comprehensive gene expression was delegated to Affymetrix Japan K.K. (Tokyo, Japan). Total RNA was extracted from the MTA‐treated RCM‐1 cells using the SV Total RNA Isolation System according to the manufacturer's instructions (Promega Corporation, Madison, WI, USA) and then shipped to the company. The relative expression data were presented as a heatmap using multiexperiment viewer software version 4.9.0 (RRID: SCR_001915, The Institute for Genomic Research, Rockville, MD, USA) [24].

### RT‐PCR

Total RNA extracted from the RCM‐1 cells was subjected to reverse transcription in a 50‐μL cocktail containing the RNA (2.5 μg), 1 × RT buffer, 0.5 mm dNTPs, 5 μm oligo(dT) primer, 40 U RNase inhibitor, and 200 U GeneAce Reverse Transcriptase (Nippon Gene Co., Ltd., Tokyo, Japan). The resulting cDNA (1 μL) was used as a template for PCR in a 20‐μL reaction mixture containing 1× Green GoTaq Flexi Buffer, 0.3 mm dNTPs, 2.0 mm MgCl_2_, 0.4 μm of each primer and 1 U GoTaq DNA polymerase (Promega Corporation). The PCR program was as follows: an initial denaturation step at 95 °C for 1 min, followed by 23–35 cycles at 98 °C for 10 s, 64–67 °C for 30 s and 72 °C for 15 s, and finally an extension step at 72 °C for 7 min. The following three sets of primers were used to amplify the target genes: 5′‐AGGAAAACTACCCAGGATGTCA‐3′ and 5′‐ATCAGGCAAAGGTGAAGGATTA‐3′ for the *CCNE2* gene; 5′‐ACCGTCACTATGGACCAGC‐3′ and 5′‐TTCAGAGCTGGACTACATCC‐3′ for *CDC25A*; and 5′‐GCACCGTCAAGGCTGAGAAC‐3′ and 5′‐TGGTGAAGACGCCAGTGGA‐3′ for the *glyceraldehyde 3‐phosphate dehydrogenase* (*GAPDH*) gene used as the internal control. The intensity of the PCR bands was estimated using imagej software (RRID: SCR_003073, http://rsb.info.nih.gov/ij/, The National Institutes of Health, Bethesda, MD, USA). After normalization with the *GAPDH* expression level, the expression of each gene was recorded as a relative value to that of the initial time (0 h, set as 1.00).

### Gene function analysis

Gene Ontology (GO) enrichment analysis and Kyoto Encyclopedia of Genes and Genomes (KEGG) pathway analysis of microarray data were performed via DAVID Bioinformatics Resources 6.8 (RRID: SCR_001881, https://david.ncifcrf.gov/home.jsp) for the differentially expressed genes in MTA‐treated RCM‐1 cells [25, 26]. The data were further applied to Gene Set Enrichment Analysis (GSEA) using GSEA v4.1.0 software (RRID: SCR_003199, http://www.broadinstitute.org/gsea/index.jsp) with 1000 gene set permutations using the ‘Diff_of_Classes’ gene‐ranking metric with the collections ‘c5.bp.v7.1symbols.gmt’ and ‘c2.cp.kegg.v7.1.symbols.gmt’ [27].

### Immunoblot analysis

The immunoblot analysis was performed according to the supplier's instructions (GE Healthcare Ltd., Buckinghamshire, UK). In brief, the RCM‐1 cell lysates were separated by 10% SDS/PAGE, and the bands were blotted onto a poly(vinylidene difluoride) membrane. The membrane was then sequentially incubated with the following solutions at room temperature: 5% skim milk in PBS containing 0.1% Tween 20 for 1 h; the primary antibodies against human CCNE2 (1 : 2000, RRID: AB_1847388; Merck KGaA), human CDC25A (1 : 100, RRID: AB_627226; Santa Cruz Biotechnology, Inc., Dallas, TX, USA) or chicken actin (1 : 2000, RRID: AB_63314; Life Technologies) for 2 h; and the horseradish peroxidase‐conjugated secondary antibodies (1 : 1500–2500) against anti‐chicken IgY for CCNE2 detection (RRID: AB_228385; Life Technologies), and anti‐mouse IgG for CDC25A and actin detection (RRID: AB_2687537; SeraCare Life Sciences, Inc., Milford, MA, USA) for 1 h. The immunoreactive CCNE2 protein was visualized on X‐ray film using the enhanced chemiluminescence detection system (GE Healthcare). In contrast, the signal of immunoreactive CDC25A was directly detected by the chemiluminescence CCD imaging system (Ez‐Capture MG; ATTO Co., Tokyo, Japan), following the visualization with WSE‐7120 EzWestLumi plus reagent (ATTO).

### Statistical analysis

Values are presented as the average dome number of each experiment. Statistical significance was determined using one‐way ANOVA, followed by Dunnett’s test for comparisons between the test and control groups, or the Tukey–Kramer honestly significant difference (HSD) test for multiple comparisons.

## Results

### Structure–activity relationship of MTPE and MTAE analogues

A structure–activity relationship analysis was performed to determine the most crucial MTPE and MTAE moiety required to induce differentiation of the RCM‐1 cells into dome tissue. Among the 37 related compounds tested, compounds **1–12** (including MTPE and MTAE) exhibited obvious dome‐inducing activity in the RCM‐1 cell culture (Table 1), whereas compounds **13–37** showed no such activity (Table 2). The dome‐inducing activity of compounds **1**–**12** was in the order of **2** > **6** > **3** > **5** > **7** > **9** > **4** (MTAE) > **8** (MTPE) > **11** > **1** > **10** > **12**. These compounds were divided into two classes of high (ED_50_ < 1 mm, compounds **2–9**) and low (ED_50_ > 1 mm, compounds **1** and **10–12**) activities. All active compounds with a methyl group at the R_1_ position belonged to the high‐activity class, except for compound **10** with its relatively lower activity (ED_50_ = 1.65 mm). Three compounds (**1**, **11** and **12**) in the low‐activity class possessed either a hydrogen atom or an ethyl group at R_1_, instead of a methyl group. Among the 12 active compounds, the number of carbon atom of methylene chains at R_2_ was limited to two or less, and that of the alkyl chains at R_3_ was three or less. Furthermore, the activity fluctuated depending on the number of carbon atom of alkyl chains there, implying an odd–even effect on the dome‐inducing capacity.

**Table 1:** Analogues of MTPE and MTAE with dome‐formation‐inducing activity. *M*_r_, relative molecular mass.
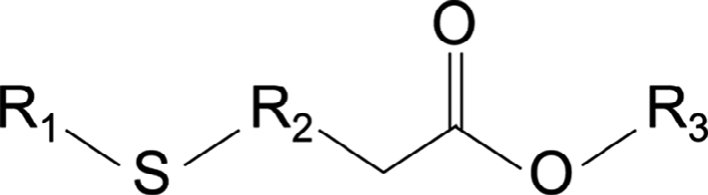

Compound no.	Name	R_1_	R_2_	R_3_	ED_50_ ^a^ (mm)	*M*_r_
1	Mercaptopropionic acid ethyl ester	H	CH_2_	C_2_H_5_	1.32	134
2	MTA	CH_3_	–	H	0.13	106
3	Methylthioacetic acid methyl ester	CH_3_	–	CH_3_	0.38	120
4	MTAE	CH_3_	–	C_2_H_5_	0.61	134
5	Methylthioacetic acid propyl ester	CH_3_	–	C_3_H_7_	0.39	148
6	3‐Methylthiopropionic acid	CH_3_	CH_2_	H	0.35	120
7	3‐Methylthiopropionic acid methyl ester	CH_3_	CH_2_	CH_3_	0.50	134
8	MTPE	CH_3_	CH_2_	C_2_H_5_	0.71	148
9	3‐Methylthiopropionic acid propyl ester	CH_3_	CH_2_	C_3_H_7_	0.52	162
10	4‐Methylthiobutyric acid	CH_3_	C_2_H_4_	H	1.65	134
11	Ethylthioacetic acid	C_2_H_5_	–	H	1.07	120
12	Ethylthioacetic acid methyl ester	C_2_H_5_	–	CH_3_	1.72	134

^a^ ED_50_ means the lowest dose needed to trigger more than 1.5‐fold increase in the dome number over the control.

**Table 2:** Analogues of MTPE and MTAE with undetectable dome‐formation‐inducing activity.*M*_r_, relative molecular mass.

Compound no.	Name	R_1_	R_2_	R_3_	ED_50_ ^a^ (mm)	*M*_r_
13	Mercaptoacetic acid	H	–	H	ND	92
14	Mercaptoacetic acid methyl ester	H	–	CH_3_	ND	106
15	Mercaptoacetic acid ethyl ester	H	–	C_2_H_5_	ND	120
16	Mercaptoacetic acid propyl ester	H	–	C_3_H_7_	ND	134
17	Mercaptoacetic acid butyl ester	H	–	C_4_H_9_	ND	148
18	Mercaptopropionic acid	H	CH_2_	H	ND	106
19	Mercaptopropionic acid methyl ester	H	CH_2_	CH_3_	ND	120
20	Mercaptopropionic acid propyl ester	H	CH_2_	C_3_H_7_	ND	148
21	4‐Methylthio butyric acid methyl ester	CH_3_	C_2_H_4_	CH_3_	ND	148
22	4‐Methylthio butyric acid ethyl ester	CH_3_	C_2_H_4_	C_2_H_5_	ND	162
23	5‐Methylthiovaleric acid	CH_3_	C_3_H_6_	H	ND	148
24	5‐Methylthiovaleric acid methyl ester	CH_3_	C_3_H_6_	CH_3_	ND	162
25	5‐Methylthiovaleric acid ethyl ester	CH_3_	C_3_H_6_	C_2_H_5_	ND	176
26	Ethylthioacetic acid ethyl ester	C_2_H_5_	–	C_2_H_5_	ND	148
27	Ethylthioacetic acid propyl ester	C_2_H_5_	–	C_3_H_7_	ND	162
28	3‐Ethylthiopropionic acid	C_2_H_5_	CH_2_	H	ND	134
29	3‐Ethylthiopropionic acid methyl ester	C_2_H_5_	CH_2_	CH_3_	ND	148
30	3‐Ethylthiopropionic acid ethyl ester	C_2_H_5_	CH_2_	C_2_H_5_	ND	162
31	4‐Ethylthiobutyric acid	C_2_H_5_	C_2_H_4_	H	ND	148
32	4‐Ethylthiobutyric acid methyl ester	C_2_H_5_	C_2_H_4_	CH_3_	ND	162
33	4‐Ethylthiobutyric acid ethyl ester	C_2_H_5_	C_2_H_4_	C_2_H_5_	ND	176
34	Propylthioacetic acid	C_3_H_7_	–	H	ND	134
35	Propylthioacetic acid methyl ester	C_3_H_7_	–	CH_3_	ND	148
36	3‐Propylthiopropionic acid	C_3_H_7_	CH_2_	H	ND	148
37	3‐Propylthiopropionic acid ethyl ester	C_3_H_7_	CH_2_	C_2_H_5_	ND	176

^a^ ED_50_ means the lowest dose needed to trigger more than 1.5‐fold increase in the dome number over the control.

**Fig. 1:**
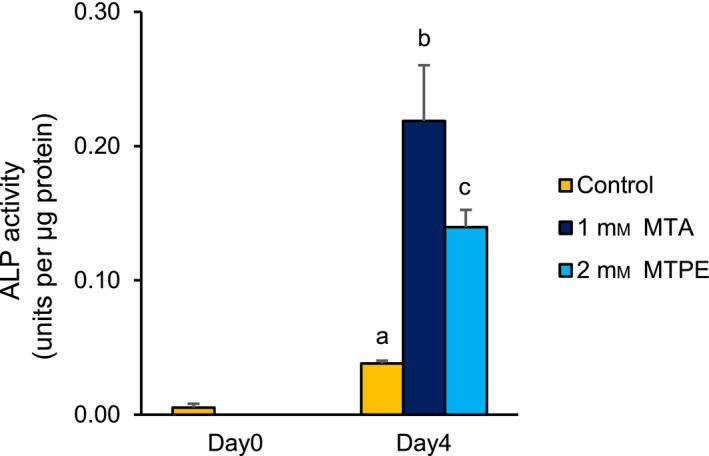
Increased ALP activity by administration of MTA or MTPE in RCM‐1 cells. RCM‐1 cells (1 × 10^5^) were seeded into 96‐well plates and cultured to near confluency. The cells were then incubated for 4 days with 1 mm MTA or 2 mm MTPE. In a control experiment, the cells were incubated without the chemicals (0.1% acetonitrile). ALP activity was measured by a spectrophotometric method using LabAssay™ ALP kit. The values of each group show mean ± SD of three experiments. Values with different letters are significantly different by one‐way ANOVA followed by Tukey–Kramer HSD test (*P* < 0.01).

MTA (compound **2**), which had the lowest molecular weight among the 37 MTPE and MTAE analogues, exhibited the most potent dome‐inducing activity (ED_50_ = 0.13 mm) (Table 1), suggesting that it was the minimum unit required to trigger dome formation. Therefore, we performed further analysis to determine whether the methylthio group in MTA is essential for inducing dome formation, by substituting oxygen, nitrogen, or carbon for the sulphur atom. As shown in Table 3, the three MTA derivatives (compounds **2a**, **2b** and **2c**) without a sulphur atom did not promote dome formation at all, confirming the significance of the methylthio group in exerting the strong induction activity. Furthermore, MTA was shown to induce ALP activity as a biochemical differentiation marker in RCM‐1 cells at even higher levels than MTPE (Fig. 1) [9]. Because MTA exhibited the most potent activity, we used it to carry out the further experiments, with its concentration set at 1 mm to secure the reproducibility of its action.

**Table 3:** MTA derivatives subjected to structure–activity relationship analysis. 
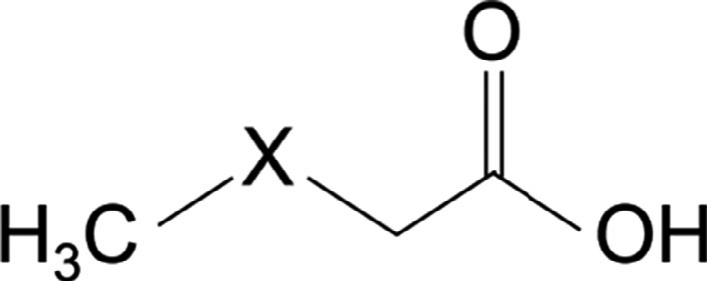

Compound no.	Name	X	ED_50_ ^a^ (mm)
**2**	MTA	S	0.13
**2a**	Methoxyacetic acid	O	ND
**2b**	Sarcosine	N	ND
**2c**	Butyric acid	C	ND

^a^ ED_50_ means the lowest dose needed to trigger more than 1.5‐fold increase in the dome number over the control.

### Microarray analysis to define candidate genes involved in MTA‐induced dome formation

**Fig. 2:**
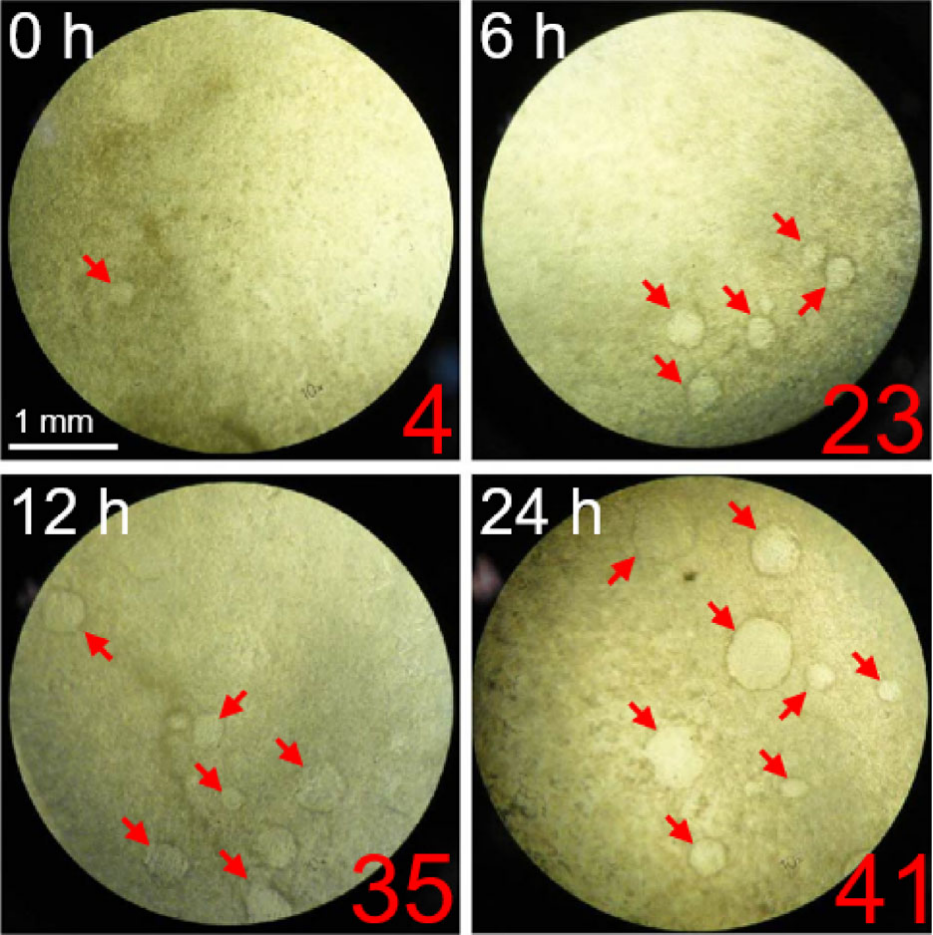
MTA enhancement of dome formation in RCM‐1 cell culture. MTA (1 mm) was applied to nearly confluent RCM‐1 cells cultured for the indicated hours, followed by additional culture in MTA‐free medium until 48 h after MTA addition. Micrographs show the domes (arrows) formed in a 24‐well plate with their numbers. Scale bar: 1 mm.

Microarray analysis is effective for finding the candidate genes involved in MTA‐induced dome formation. To conduct this analysis, we had previously determined the adequate duration of MTA treatment for RCM‐1 differentiation induction. As shown in Fig. 2, the dome number was increased nearly six times compared with that of the mock control when RCM‐1 cells were treated with MTA for 6 h and then cultured in MTA‐free medium until 48 h, allowing the domes to grow enough to be observed under a microscope. The number was further increased with prolongation of the MTA treatment for 12 and 24 h. Thus, MTA treatment for 6–24 h was considered to be sufficient to trigger dome formation. Therefore, for the microarray analysis, we treated RCM‐1 cells with MTA for 6, 12 or 18 h, and then cultured the cells in the vehicle medium until 24 h, at which time the dome tissues were mostly undetectable.

**Fig. 3:**
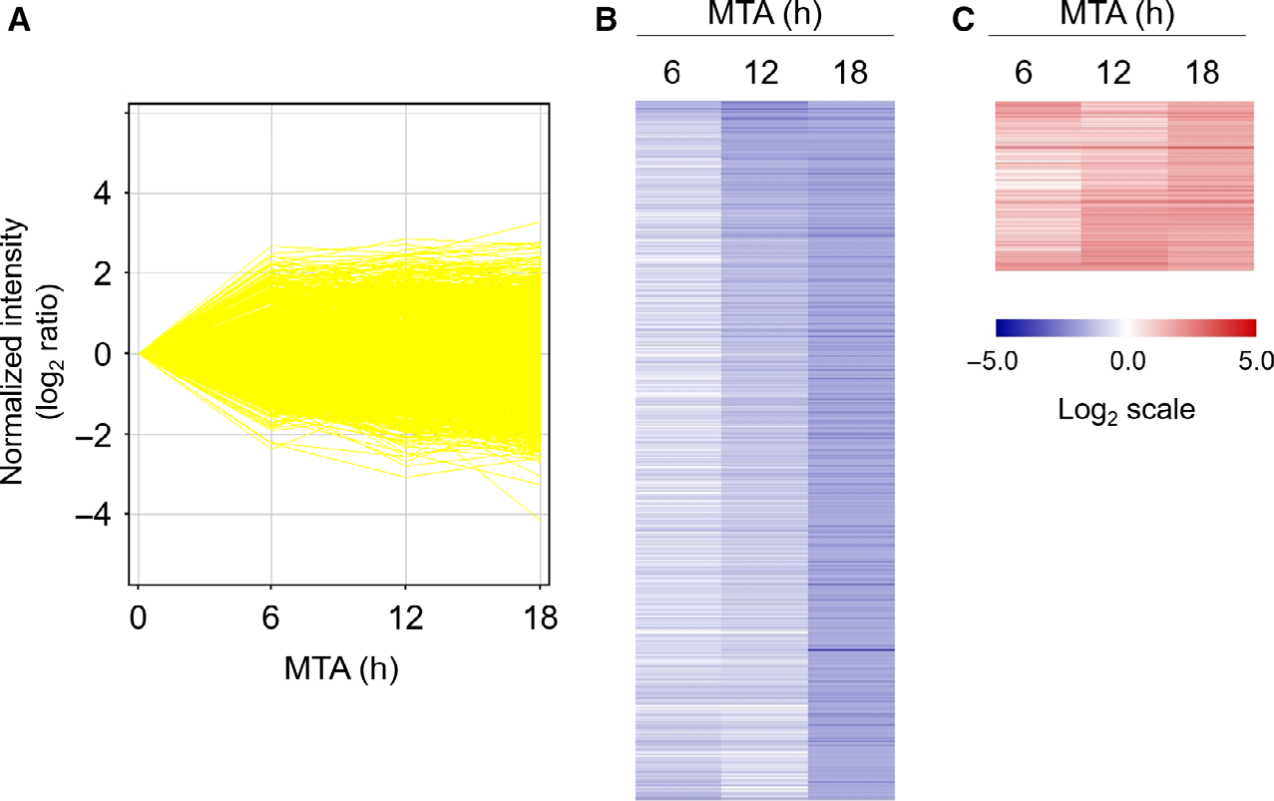
Microarray analysis of gene expression profiles in MTA‐treated RCM‐1 cells. RCM‐1 cells were treated with 1 mm MTA for the indicated times and then cultured in medium without the chemical for up to 24 h. The total RNA isolated from the cells was then subjected to microarray analysis. The gene expression profiles are depicted in two different manners. In the line graph (A), each line indicates the expression patterns of individual genes that corresponded to 20 954 probes with fluorescence intensities over the threshold. Heatmaps (B and C) of genes expressed differentially upon MTA treatment are shown in the range from lower (blue) to higher expression (red), the levels of which reached less than one‐fourth (log_2_ ratio < −1.5) (B) or more than 4‐fold (log_2_ ratio > 1.5) (C) at 18 h relative to the initial values.

**Fig. 4:**
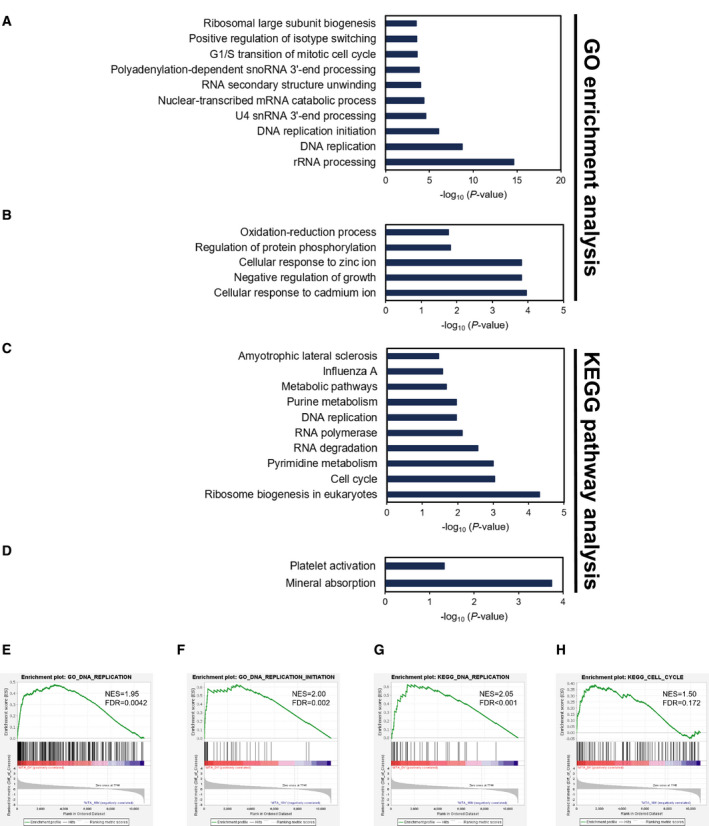
Gene function analyses of differentially expressed genes in MTA‐treated RCM‐1 cells. GO enrichment analysis (A, B) and KEGG pathway analysis (C, D) were performed using DAVID on the 537 (565 probes) and 117 genes (138 probes) that showed significantly reduced (log_2_ ratio < −1.5) and increased (log_2_ ratio > 1.5) expression levels after MTA treatment, respectively. GSEA was further performed to confirm the enrichment of GO terms and KEGG pathways via DAVID. Graph (A) shows the top 10 GO terms among 64 enriched terms in the ‘biological process’ class for the down‐regulated genes, whereas graph (B) shows all five GO terms for the up‐regulated genes. Graphs (C) and (D) show, respectively, all 10 enriched KEGG pathways for the down‐regulated genes and all two pathways for the up‐regulated genes. The GO terms and KEGG pathways with *P* < 0.05 were listed in decreasing order of the *P* value. (E, F, G, H) Graphs show GSEAs having the significant enrichment of the gene sets involved in DNA replication (E; GO), DNA replication initiation (F; GO), DNA replication (G; KEGG) and cell cycle (H; KEGG). FDR, false discovery rate; NES, normalized enrichment score.

The GeneChip Human Genome U133 Plus 2.0 Array (Affymetrix) was used to comprehensively identify the genes with altered expression levels upon MTA treatment. As depicted in Fig. 3A, among the entire set of 54 675 probes applied, 20 954 probes were detected, of which 537 (log_2_ ratio < −1.5) and 117 (log_2_ ratio > 1.5) genes were strongly down‐regulated and up‐regulated by the 18‐h MTA treatment, respectively, relative to the mock control levels (Fig. 3B,C). These genes were then applied to the GO enrichment analysis via DAVID. For the 537 down‐regulated genes, 64 terms (*P* < 0.05) were selected in the GO category of ‘biological process’, which included six terms related to cell‐cycle control to which 45 of the down‐regulated genes belonged (Table 4). Furthermore, three of these GO terms, namely, ‘DNA replication (*P*‐value = 1.9E‐9, the second place)’, ‘DNA replication initiation (*P*‐value = 8.7E‐7, the third)’ and ‘G1/S transition of mitotic cell cycle (*P*‐value = 2.3E‐4, the eighth)’, were ranked in the top 10 GO terms (Fig. 4A), although the first place was yielded to ‘rRNA processing (*P*‐value = 2.3E‐15)’. In contrast, only five GO terms were annotated for the 117 up‐regulated genes, of which no cell‐cycle‐related term was recovered (Fig. 4B). KEGG analysis via DAVID selected 10 pathways for the down‐regulated gene set, among which the ‘cell cycle (*P*‐value = 9.1E‐4)’ and ‘DNA replication (*P*‐value = 1.1E‐2)’ pathways were included (Fig. 4C), whereas no cell‐cycle‐related pathway was found for the up‐regulated gene set (Fig. 4D). Furthermore, GSEA revealed the enrichment of the ‘DNA replication (GO & KEGG)’, ‘DNA replication initiation (GO)’ and ‘cell cycle (KEGG)’ categories, supporting the analyses via DAVID (Fig. 4E–H). The results imply that MTA‐induced dome formation is linked with the down‐regulated genes rather than the up‐regulated ones.

**Table 4:** Enriched GO terms related to cell‐cycle control for the genes down‐regulated by MTA.

GO term	*P*‐value	Gene no.	Gene symbol (probe set ID)
DNA replication (GO:0006260)	1.9E−9	21	*ATR* (209902_at), *BARD1* (242655_at, 205345_at), *POLA2* (204441_s_at), *POLE3* (208828_at), *GINS2* (221521_s_at), *GINS3* (218719_s_at), *RBM14* (204178_s_at), *RECQL4* (213520_at), *TIPIN* (219258_at), *CDC25A* (1555772_a_at), *CDC6* (203967_at, 203968_s_at), *CINP* (217598_at), *DTL* (218585_s_at, 222680_s_at), *MCM10* (220651_s_at, 223570_at), *MCM9* (219673_at), *MCM3* (201555_at), *MCM4* (222037_at, 212141_at), *MCM5* (216237_s_at), *NFIB* (213033_s_at), *TBRG1* (225818_s_at), *TWNK* (218590_at)
DNA replication initiation (GO:0006270)	8.7E−7	9	*POLA2* (204441_s_at), *CDC6* (203967_at, 203968_s_at), *CCNE1* (213523_at), *CCNE2* (205034_at, 211814_s_at), *MCM10* (220651_s_at, 223570_at), *MCM3* (201555_at), *MCM4* (222037_at, 212141_at), *MCM5* (216237_s_at), *PURA* (229167_at)
G1/S transition of mitotic cell cycle (GO:0000082)	2.3E−4	11	*POLA2* (204441_s_at), *SKP2* (210567_s_at), *CDC25A* (1555772_a_at), *CDC6* (203967_at, 203968_s_at), *CUL2* (203078_at), *CCNE1* (213523_at), *CCNE2* (205034_at, 211814_s_at), *MCM10* (220651_s_at, 223570_at), *MCM3* (201555_at), *MCM4* (222037_at, 212141_at), *MCM5* (216237_s_at)
Regulation of cell cycle (GO:0051726)	3.9E−3	10	*E2F2* (228361_at), *JUN* (201466_s_at), *SKP2* (210567_s_at), *CDC25A* (1555772_a_at), *CCNE1* (213523_at), *CCNE2* (211814_s_at, 205034_at), *CDK12* (225691_at), *CDKL1* (235512_at), *DTL* (218585_s_at, 222680_s_at), *PES1* (202212_at)
Cell proliferation (GO:0008283)	2.2E−2	17	*CDV3* (213548_s_at), *E2F8* (219990_at), *PAK1IP1* (218886_at), *POLR3G* (206653_at), *SKP2* (210567_s_at), *WDR12* (241687_at), *AREG* (205239_at), *BYSL* (203612_at), *CDC25A* (1555772_a_at), *MCM10* (223570_at, 220651_s_at), *PES1* (202212_at), *PA2G4* (208676_s_at), *RAC1* (1567457_at), *SLC29A2* (204717_s_at), *TRIM27* (210541_s_at), *UHRF1* (225655_at), *MYC* (202431_s_at)
Positive regulation of DNA endoreduplication (GO:0032877)	4.9E−2	2	*PRELID1* (224232_s_at), *MYC* (202431_s_at)

Interestingly, 13 MTA‐down‐regulated genes related to cell‐cycle control were shared between the GO terms and the KEGG pathways (Table 5). Besides, eight of them are annotated to the GO term ‘G1/S transition of mitotic cell cycle’. Among those genes, *CCNE2* (−6.61‐fold, 0 versus 18 h) and *CDC25A* (−5.47‐fold, 0 versus 18 h) were the most strongly down‐regulated by MTA, followed by *minichromosome maintenance complex component 4* (*MCM4*) (−4.95‐fold, 0 versus 18 h) and *cell division cycle 6* (*CDC6*) (−4.70‐fold, 0 versus 18 h) (Table 5). CCNE2 and CDC25A proteins are known to act cooperatively at a checkpoint in the G1 phase, which is thought to be a gateway from the cell division cycle to cell differentiation [28, 29]. Thus, we focused on these two genes and further examined their relationship with MTA‐induced dome formation.

**Table 5:** MTA down‐regulated genes related to cell‐cycle control.

Gene symbol	Probe set ID	Fold change^a^			GO term	KEGG pathway
		6 h	12 h	18 h		
*CCNE2*	205034_at	1.07	−2.43	−6.61	DNA replication initiation, G1/S transition of mitotic cell cycle, regulation of cell cycle	Cell cycle
	211814_s_at	−1.14	−2.97	−4.52		
*CDC25A*	1555772_a_at	−1.26	−4.56	−5.47	DNA replication, G1/S transition of mitotic cell cycle, regulation of cell cycle, cell proliferation	Cell cycle
*MCM4*	212141_at	−1.50	−1.65	−4.95	DNA replication, DNA replication initiation, G1/S transition of mitotic cell cycle	DNA replication, cell cycle
	222037_at	−1.16	−1.65	−3.31		
*CDC6*	203968_s_at	−1.04	−1.66	−4.70	DNA replication, DNA replication initiation, G1/S transition of mitotic cell cycle	Cell cycle
	203967_at	1.06	−1.64	−4.27		
*E2F2*	228361_at	−1.57	−1.88	−4.27	Regulation of cell cycle	Cell cycle
*SKP2*	210567_s_at	−1.64	−1.83	−4.05	Regulation of cell cycle, cell proliferation	Cell cycle
*CCNE1*	213523_at	−1.29	−2.57	−3.98	DNA replication initiation, G1/S transition of mitotic cell cycle, regulation of cell cycle	Cell cycle
*POLA2*	204441_s_at	−1.21	−1.60	−3.64	DNA replication, G1/S transition of mitotic cell cycle	DNA replication
*MCM3*	201555_at	−1.32	−1.84	−3.29	DNA replication, DNA replication initiation, G1/S transition of mitotic cell cycle	DNA replication, cell cycle
*MCM5*	216237_s_at	−1.11	−1.67	−3.29	DNA replication, DNA replication initiation, G1/S transition of mitotic cell cycle	DNA replication, cell cycle
*MYC*	202431_s_at	−2.19	−2.43	−2.96	Cell proliferation, positive regulation of DNA endoreduplication	Cell cycle
*POLE3*	208828_at	−1.83	−2.46	−2.90	DNA replication	DNA replication
*ATR*	209902_at	−1.55	−2.02	−2.85	DNA replication	Cell cycle

^a^ Fold change means the fluctuation in gene expression relative to the initial value (= 1.00) for each gene.

### Validation of the microarray data by RT‐PCR and immunoblotting

**Fig. 5:**
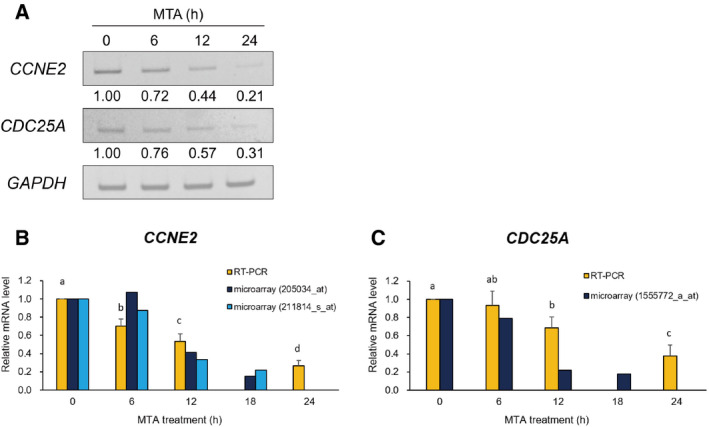
RT‐PCR verification of the MTA‐mediated down‐regulation of *CCNE2* and *CDC25A*. RT‐PCR was performed to confirm the reduction in *CCNE2* and *CDC25A* mRNA expression that was screened in the microarray analysis. After MTA treatment for the indicated hours, the total RNA was subjected to RT‐PCR. The electrophoretically separated PCR bands were stained with ethidium bromide, and representative gel images (A) of three replicates are shown. The numbers under the photographs show the fluorescence intensity of each band relative to an initial value (= 1.00), which was calculated after normalization against *GAPDH* as a reference. The bar graphs show the fluctuations of the *CCNE2* (B) and *CDC25A* (C) expression levels obtained independently by the RT‐PCR and microarray analyses. The relative mRNA levels determined by RT‐PCR represent mean ± SD of three experiments. Values with different letters are significantly different by one‐way ANOVA followed by Tukey–Kramer HSD test (*P* < 0.01). The characters in parentheses indicate the probe set ID of the microarray.

**Fig. 6:**
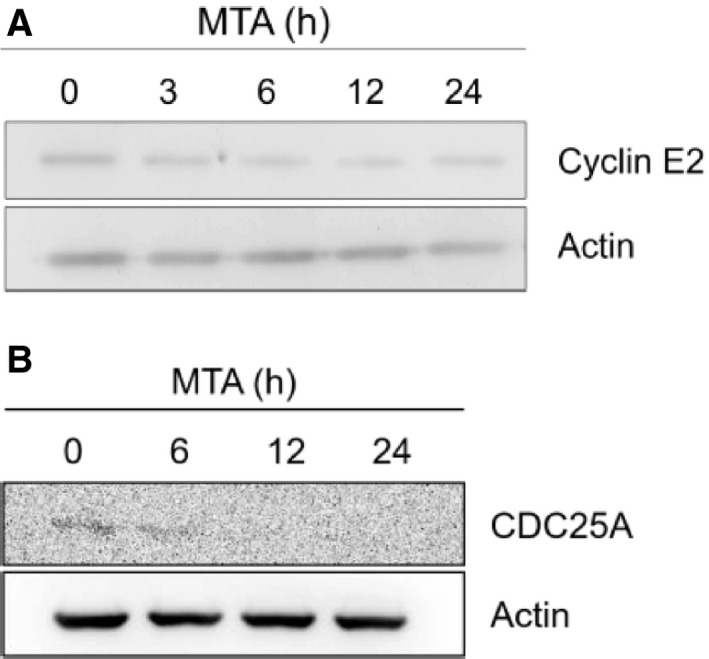
MTA‐induced reduction of the CCNE2 and CDC25A proteins in RCM‐1 cells. Following MTA treatment for the indicated hours, proteins extracted from the cells were applied to immunoblotting with the respective amounts of 3.75 µg (per each lane) for detecting CCNE2 (A) and 30 µg for CDC25A (B). The photographs show the immunologically detected proteins together with actin as a reference.

Because the microarray analysis indicated that MTA down‐regulated *CCNE2* and *CDC25A* significantly, we performed RT‐PCR analysis to confirm whether the two genes are indeed down‐regulated by MTA. As expected, the mRNA levels of *CCNE2* and *CDC25A* were reduced upon MTA treatment in a time‐dependent manner (Fig. 5A). Quantitative measurement of the PCR products further disclosed that the time dependency of the *CCNE2* mRNA reduction by MTA was quite similar between the microarray and RT‐PCR results (Fig. 5B). In contrast, *CDC25A* mRNA level was more gradually declined, compared with that of microarray (Fig. 5C). Taken together, the results confirmed that both *CCNE2* and *CDC25A* were down‐regulated by MTA. Furthermore, the amount of CCNE2 and CDC25A proteins was also reduced by MTA in a time‐dependent manner (Fig. 6).

### Enhanced dome formation by cell‐cycle inhibitors in RCM‐1 cell culture

To examine whether CCNE2 and CDC25A are directly involved in MTA‐induced dome formation, we next conducted a pharmacological analysis using purvalanol A, an inhibitor of cyclin‐dependent kinases (CDKs; CDK1 and CDK2) [30], and NSC95397, an inhibitor of CDC25 dual‐specificity phosphatases (CDC25A, CDC25B and CDC25C) [31]. Consequently, purvalanol A and NSC95397 were expected to inactivate the CCNE2–CDK2 complex and CDC25A phosphatase in the G1 phase, respectively. As shown in Table 6, the average dome number was increased by approximately 1.3‐ to 2.1‐fold relative to the mock control number when applying purvalanol A in the range of 2.8–90 nm. In most cases (with some exceptions), the fold increase was beyond our criterion of 1.5‐fold that was used to identify phytochemicals from Katsura‐uri melon with dome‐formation‐inducing activity [9]. Purvalanol A treatment at 45 and 90 nm showed the strongest effects on dome formation compared with the other concentrations. The average dome number in RCM‐1 cells treated with NSC95397 in the range of 0.9–30 µm was also increased approximately 1.8‐ to 3.5‐fold relative to the mock control levels. NSC95397 treatment at 1.9 and 15 µm showed the strongest effects on dome formation. Thus, NSC95397 apparently showed a much more potent dome‐inducing activity than purvalanol A. Indeed, the increase (3.5‐fold) in dome number caused by 15 μm NSC95397 was approximately 1.7 times higher than that of 45 and 90 nm purvalanol A (2.0‐ and 2.1‐fold, respectively). However, their dose ranges were mutually different by more than 2 orders of magnitude. Recently, we applied MTA (1 mm) together with either NSC95397 (15 µm) or purvalanol A (45 nm) to RCM‐1 cells to examine a pharmacological interaction. As depicted in Table 7, when coapplying MTA and NSC95397, an average dome number (6.2‐fold) was increased, compared with the single treatment of each chemical, MTA (4.8‐fold) and NSC95397 (3.7‐fold). In contrast, purvalanol A addition did not affect the dome number induced by MTA. The result suggests that interaction between MTA and NSC95397 is ‘additive’ or ‘partially antagonistic’, but not ‘synergistic’, presumably contradicting a notion that MTA induces dome formation in a molecular pathway independent of NSC95397, although it remains unknown for the interaction between MTA and purvalanol A [32].

**Table 6:** Dome formation triggered by two cell‐cycle inhibitors, purvalanol A and NSC95397.

Chemical	Average dome number^a^	Fold increase^b^
MTA (mm)		
1	16.2*	5.4
Purvalanol A (nm)		
2.8	4.8	1.6
5.6	5.1	1.7
11.3	3.8	1.3
22.5	5.2	1.7
45	6.1	2.0
90	6.2	2.1
NSC95397 (µm)		
0.9	6.3	2.1
1.9	9.8*	3.3
3.8	5.4	1.8
7.5	6.2	2.1
15	10.4*	3.5
30	7.6*	2.5

^a^ The value is the average dome number in the well of a 96‐well plate of 12 tests.

^b^ Fold increase means the relative increase in dome number over that of the mock treatment (= 1.0).

** *P* < 0.05.

* *P* < 0.01 (one‐way ANOVA followed by Dunnett’s test).

**Table 7:** Dome formation triggered by coapplication of MTA with Purvalanol A or NSC95397.

Chemical	Average dome number^a^	Fold increase^b^	Group^c^
DMSO	4.1	1.0	A
1 mm MTA (1 mm)	19.6	4.8	C
Purvalanol A (45 nm)	5.7	1.4	A
NSC95397 (15 µm)	15.5	3.7	B
Purvalanol A + MTA	18.4	4.5	C
NSC95397 + MTA	25.6	6.2	D

^a^ The value is the average number of domes formed in a well of a 96‐well plate (*n* = 31 or 32).

^b^ Fold increase means the relative increase in dome number over that of the mock treatment (= 1.0).

^c^ The same capital letter indicates no significant difference (*P* < 0.01, determined by one‐way ANOVA followed by Tukey–Kramer HSD test).

## Discussion

This study clarified that among the analogue compounds of MTPE and MTAE, which were previously isolated from Katsura‐uri melon [22], MTA is the basic structural unit needed to exert the potent dome‐formation‐inducing activity in RCM‐1 human colorectal cancer cells. In addition, we found that MTA triggered the down‐regulation of a large number of genes, including those acting in cell‐cycle control and DNA replication. Herein, we discuss the chemical structures indispensable for the dome‐inducing activity and the possible involvement of two down‐regulated genes (i.e. *CDC25A* and *CCNE2*) in MTA‐induced dome formation.

The determination of the critical moiety in MTPE and MTAE analogues for inducing dome formation would be helpful toward elucidating the underlying mechanisms of the process. Through careful structure–activity relationship analyses, we discovered several features required for dome formation, as described later. First, we uncovered that a methyl group at the R_1_ position was essential for inducing a strong dome‐inducing activity because this group was observed in all compounds (**2–9**) with high dome‐formation‐inducing activity (Table 1). In fact, the replacement of the methyl group with any other group caused a loss in the activity (Tables 1 and 2), although there were some exceptions: the compounds (**1**, **11**, and **12**) with either a hydrogen atom or an ethyl group had marginal activity. Second, we found that the functional group at R_2_ was also important for inducing dome formation in cooperation with the methyl group at R_1_, because the high‐activity compounds (**2–9**) with a methyl group at R_1_ always had either a methylene group or nothing at R_2_. Moreover, substitution of the methylene group with either an ethylene or a trimethylene group caused the reduction or complete loss of the dome‐inducing activity. Thus, a methyl group at R_1_, together with either a methylene group or nothing at R_2_, is presumably necessary for the strong dome‐formation‐inducing activity. Third, the functional group at R_3_ likely determined the strength of the dome‐inducing activity because a hydrogen atom at R_3_ allowed several compounds (**2**, **6** and **11**) to have an activity higher than those with an alkyl group at R_3_ (**3–5**, **7–9** and **12**). In addition, an odd–even effect was observed regarding the alkyl group at R_3_; the compounds (**3**, **5**, **7** and **9**) with odd‐numbered alkyl chains had a higher activity than those (**4** and **8**) with even‐numbered alkyl chains. These results imply that the hydrogen atom and alkyl chain at R_3_ are considerably important for inducing dome formation, and that the length and parity of the alkyl chain also govern the activity strength. Finally, the sulphur element was disclosed to be essential for inducing dome formation because its replacement with oxygen, nitrogen or carbon caused a complete loss in the dome‐inducing activity (compounds **2a**, **2b** and **2c**) (Table 3). We concluded that MTA was the basic structural unit required to exert the most potent dome‐formation‐inducing activity among that of the 37 MTPE and MTAE analogues tested.

It is reasonable to hypothesize that MTA leads to dome formation by affecting and/or restoring partly aberrant gene expression programs in the RCM‐1 cancer cells, because deregulated gene expression is a general hallmark of cancers [33]. Consistent with this notion, our microarray analysis demonstrated that MTA certainly affected the expression of numerous genes (654), of which 82.1% (537) were down‐regulated and the remaining 17.9% (117) were up‐regulated (Fig. 3). This may suggest that MTA‐induced dome formation is more tightly linked with global down‐regulation of genes rather than their up‐regulation, because there was a strong bias in the number between down‐regulated and up‐regulated genes. More interestingly, 45 down‐regulated genes were classified into the six GO terms that are related to cell division and proliferation (Table 4). Moreover, more than half (29) of these genes are annotated to roles in early cell‐cycle events, such as G1/S transition (4), DNA replication (14) and both (7). The G1 phase is believed to be a gateway for the cell fate determination leading to quiescence, differentiation, senescence and apoptosis [28, 29]. Thus, some components acting in the G1 and S phases could be associated with MTA‐induced dome formation because their dysfunction potentially causes G1 arrest. CDC25A phosphatase and CCNE2, the key regulators of G1 progression, are of great interest because they were most strongly down‐regulated by MTA among the genes involved in cell‐cycle control (Table 5 and Fig. 5A).

**Fig. 7:**
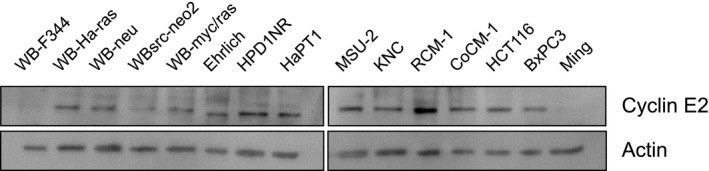
CCNE2 accumulation in various mammalian cells. Immunoblotting was performed to examine CCNE2 accumulation in different nontransformed and transformed mammalian cell lines. The following cell lines were used: WB‐F344, WB‐F344 rat liver epithelial cells; WB‐Ha‐ras, v‐Ha‐ras‐transformed WB; WB‐neu, neu‐transformed WB; WBsrc‐neo2, v‐src‐transformed WB; WB‐myc/ras, myc/ras‐transformed WB; Ehrlich, Ehrlich ascite tumor cells; HPD1NR, transplantable pancreatic ductal cancer cells; HaPT1, hamster pancreatic cancer cells; MSU‐2, human foreskin fibroblast cells; KNC, human well‐differentiated colon cancer cells; RCM‐1, human well‐differentiated rectal cancer cells; CoCM‐1, human moderately differentiated colon cancer cells; HCT116, human poorly differentiated colon cancer cells; BxPC3 and Ming, human pancreatic cancer cells.

CDC25A dual‐specificity phosphatase drives G1 progression through CDK2 activation by removing the inhibitory phosphate groups [34, 35]. Meanwhile, cyclin E forms a complex with CDK2 to regulate the kinase activity in a phase‐dependent manner. The complex reportedly regulates G1/S transition through phosphorylation of the target proteins, such as retinoblastoma protein, SMAD family member 3, CREB‐binding protein/p300 and E2F transcription factor 5 [36, 37, 38, 39]. Although our knowledge of cyclin E has been mostly obtained from the studies on cyclin E1, one of the two E‐type cyclin proteins (i.e. E1 and E2) in humans, CCNE2 likely plays a similar role in this process [40]. Accordingly, CDC25A and CCNE2 may function collaboratively on G1 progression because they share CDK2 as the target and the partner, respectively. Moreover, the two proteins have been described to be highly accumulated in different cancers, such as CDC25A in hepatocellular, lung, laryngeal and head and neck squamous cell cancers [41, 42, 43, 44], and CCNE2 in breast and gastric cancers [45, 46]. In fact, our immunoblot assay uncovered the high accumulation of CCNE2 in RCM‐1 cells compared with other cell lines (Fig. 7).

The question is whether MTA induces dome formation through the down‐regulation of *CDC25A* or *CCNE2*, or both. Our pharmacological analysis somewhat supported this hypothesis because the number of domes in the RCM‐1 cell culture was adequately increased independently of MTA when treated with a CDC25 inhibitor (NSC95397) at the concentrations of 0.9–30 µm (Table 6). Furthermore, the simultaneous application of MTA (1 mm) with NSC95397 (15 µm) indicated that their pharmacological interaction on the dome formation is rather ‘additive’ or ‘partially antagonistic’, but not ‘synergistic’ (Table 7), thereby not supporting that the two chemicals may affect the events in the different pathways connected to the same goal [32]. NSC95397 reportedly inhibits the catalytic activities of three human CDC25 phosphatases (A, B and C) [31]. Although this inhibitor has so far been thought to cause G2/M arrest by affecting mostly CDC25B and CDC25C [31, 47, 48], it might bring about G1 arrest in our case by inhibiting the CDC25A phosphatase acting in G1/S transition, thereby leading to dome formation. In addition, NSC95397 (20 µm) was previously shown to accelerate CDC25A degradation via the proteasome pathway in two human prostate cancer cell lines, PC‐3 and LNCap [49]. Therefore, it is reasonable to consider that MTA promotes dome formation by down‐regulating *CDC25A*, which may then lead to decreases in the CDC25A protein content and activity (Fig. 6). However, careful interpretation is needed, because the inhibition of the other CDC25 phosphatases (B and C) by NSC95397 might indirectly affect dome formation as well. On the contrary, purvalanol A, an inhibitor of CDK1 and CDK2 [30], did not sufficiently support the aforementioned hypothesis, although we expected that this chemical and the MTA‐induced *CCNE2* down‐regulation would lead to similar consequences, given that CDK2 and CCNE2 form a complex in the G1 phase [50, 51]. Indeed, purvalanol A did enhance dome formation at the concentrations of 2.8–90 nm without MTA, but its effect was much lower than that of NSC95397 (Table 6). A possible explanation for this ambiguous result is that purvalanol A suppresses the roles of other cyclin/CDK complexes, such as cyclin B/CDK1, in the M phase and cyclin A/CDK2 in the S and G2 phases, aside from cyclin E (either E1 or E2)/CDK2 in the G1 phase [30], thus disturbing different aspects of cell‐cycle control, although other explanations cannot be excluded. CCNE2 still remains a putative component in MTA‐induced dome formation because cyclin E has been proved to be a target of the CDC25A phosphatase in the G1 phase [52].

Altogether, the results of our study indicate that MTA commits RCM‐1 cancer cells toward their differentiation into dome tissue via the down‐regulation and dysfunction of CDC25A and possibly of CCNE2, the key cell‐cycle regulators in the G1 phase. However, there still remains research to be addressed for the elucidation of the molecular mechanisms behind MTA‐induced dome formation, although the findings in this study provided an important first step toward this goal. For instance, intensive pharmacological analysis with different cell‐cycle inhibitors and molecular genetic work to knock out (or knock down) the earlier candidates should be conducted to solidify and more clearly explain our current findings. In addition, application of MTA on colon cancer cell lines other than RCM‐1 is also of interest to determine its universality regarding the dome‐inducing activity. These studies would provide further evidence to elucidate more clearly the mechanisms of dome formation by MTA, which may then give a hint(s) to develop the MTA‐based differentiation‐inducing substances, as well as other novel therapeutic ways for colorectal cancer in the future.

## Conflict of interest

The authors declare no conflict of interest.

## Author contributions

YN, TM and SO designed the whole study. MK, SW, YN, KK, FH and KI conducted and interpreted the structure–activity relationship analysis. MK, SW, ST, YO, YN and SO conducted the global gene expression study and analyzed the obtained data. MK, AF, KT and YN carried out the immunoblotting analysis. MK, AS, SW and TN performed the pharmacological study and analyzed the data. MK, YN and SO wrote the manuscript. All authors read and approved the final manuscript.

## Data Availability

Gene expression data are available in the GEO database under the accession number GSE139279. Data will also be available from the corresponding author upon reasonable request.
